# Effects of Melatonin on Glycemic Variability in Type 2 Diabetes Mellitus: Protocol for a Crossover, Double-Blind, Placebo-Controlled Trial

**DOI:** 10.2196/47887

**Published:** 2023-08-16

**Authors:** Wagner Martorina, Almir Tavares

**Affiliations:** 1 Institute of Biological Sciences Federal University of Minas Gerais Belo Horizonte Brazil; 2 Department of Mental Health Faculty of Medicine Federal University of Minas Gerais Belo Horizonte Brazil

**Keywords:** type 2 diabetes mellitus, glycemic control, melatonin, randomized controlled trial, cross-over studies, T2DM, glucose, glycemic variability, circadian rhythm

## Abstract

**Background:**

Glycemic variability is recognized as a significant factor contributing to the development of micro- and macrovascular complications in individuals with type 2 diabetes mellitus (T2DM). Numerous studies have shown that melatonin, a hormone involved in regulating various biological rhythms, including those related to glucose regulation, such as hunger, satiety, sleep, and circadian hormone secretion (ie, cortisol, growth hormone, catecholamines, and insulin), is deficient in individuals with T2DM. This raises an important question: Could melatonin replacement potentially reduce glycemic variability in these patients? This warrants investigation as a novel approach to improving glycemic control and reducing the risk of complications associated with T2DM.

**Objective:**

We aimed to investigate whether melatonin replacement in individuals with T2DM who supposedly have melatonin deficiency can positively impact the regulation of insulin secretion rhythms and improve insulin sensitivity, ultimately resulting in a reduction in glycemic variability.

**Methods:**

This study will use a crossover, randomized, double-blind, placebo-controlled trial design. Patients with T2DM in group 1 will receive 3 mg of melatonin at 9:00 PM in the first week, undergo a washout period in the second week, and receive a placebo in the third week (melatonin-washout-placebo). Group 2 will be randomized to receive a placebo-washout-melatonin sequence (3 mg). Capillary blood glucose levels will be measured at 6 different times before and after meals during the last 3 days of the first and third weeks. The study aims to compare the mean differences in blood glucose levels and the coefficient of glycemic variability in patients receiving melatonin and placebo during the first and third weeks. After analyzing the initial results, the number of needed patients will be recalculated. If the recalculated number is higher than 30, new participants will be recruited. Thirty patients with T2DM will be randomized into the 2 groups: melatonin-washout-placebo or placebo-washout-melatonin.

**Results:**

Participant recruitment took place between March 2023 to April 2023. In all, 30 participants were eligible and completed the study. We expect that patients will show different glycemic variability on the days they receive placebo or melatonin. Studies on melatonin and glycemic control have shown both positive and negative results. We hope that there will be a positive outcome regarding glycemic variability (ie, a reduction in glycemic variability), as melatonin has a well-described chronobiotic effect in the literature.

**Conclusions:**

This study aims to determine whether melatonin supplementation can effectively reduce glycemic variability in patients with T2DM. The crossover design is necessary due to the multiple variables involved in the circadian variations of glucose, including diet, physical activity, sleep parameters, and pharmacological treatments. The relatively low cost of melatonin and its potential role in reducing the severe complications associated with T2DM have motivated this research effort. Furthermore, the indiscriminate use of melatonin in current times makes conducting this study essential to evaluate the effect of this substance in patients with T2DM.

**Trial Registration:**

Brazilian Registry of Clinical Trials RBR-6wg54rb; https://ensaiosclinicos.gov.br/rg/RBR-6wg54rb

**International Registered Report Identifier (IRRID):**

DERR1-10.2196/47887

## Introduction

Despite recent advances in medical technology and treatments for type 2 diabetes mellitus (T2DM), the prevalence of this disease is still increasing exponentially worldwide [1]. According to the International Diabetes Federation, the number of adults with T2DM globally was expected to rise to 463 million in 2019 and 700 million by 2045, with a significant impact on public health, society, and the economy [2]. Effective treatment of T2DM is crucial in avoiding complications and improving overall health outcomes for patients. In recent years, the management of T2DM has evolved significantly due to the discovery of new therapeutic targets and the development of new drugs with different mechanisms of action [3]. Additionally, nondrug interventions that impact glycemic control have become increasingly important in the management of T2DM [4,5]. Dietary modification, physical activity, weight management, and stress management have long been recommended. In addition to lifestyle modifications and medication, the role of good quality sleep in managing T2DM has become increasingly recognized by health care professionals. The American Diabetes Association’s 2017 position statement underscored the significance of adequate sleep in the management of T2DM and brought attention to the detrimental effect that sleep disturbances can have on glycemic control [6]. Melatonin, a hormone linked to the circadian rhythm, may be the pathophysiological link between sleep changes and glycemic control [7]. This hormone synchronizes our waking, sleeping, hunger, and satiety times, as well as other biological functions, such as insulin secretion. Previous studies found that melatonin production is reduced in T2DM [8-10]. While some papers have suggested that melatonin replacement may have benefits for glycemic control, the results of these studies have been conflicting, with some studies not showing significant benefits [11-14]. How can such differences be explained? The impact of melatonin on glycemic control may depend on several factors, including the timing and duration of use, as well as individual genetic variations, such as *MT2* melatonin receptor polymorphisms [15,16] Mutations in the *MT2* receptor gene have been associated with altered glucose metabolism and insulin sensitivity and may impact the effectiveness of melatonin supplementation in improving glycemic control. Some studies have suggested that individuals with *MT2* receptor mutations may experience an increase in glycohemoglobin levels following melatonin use, unlike those without this mutation.

Assessing variations in glucose throughout the day, instead of just the average glycohemoglobin level, is an important aspect of investigating the potential impact of melatonin on glycemic control. In this work, we will not evaluate the role of melatonin in average glucose (glycohemoglobin) level, but on variations in glucose level throughout the day. Our proposal is to determine whether melatonin influences the circadian rhythm of glucose in patients with T2DM. Currently, no study has evaluated whether melatonin has such a role.

Glycemic variations throughout the day depend on central and peripheral control of insulin secretion and sensitivity. Previous studies have linked the action of melatonin on *MT1* and *MT2* receptors to insulin secretion and sensitivity [17]. Our hypothesis is that melatonin replacement in people with T2DM who are melatonin deficient may have a positive role in the regulation of insulin, cortisol, and the rhythms of other secretions. If this hypothesis is correct, melatonin replacement may reduce the range of blood glucose variation throughout the day. It has already been demonstrated that wide circadian glucose variation in patients with diabetes mellitus can be a stress and inflammation factor and lead to chronic complications of the disease that occur when glycohemoglobin is elevated [18,19]. Thus, this study asks, Could melatonin replacement in these melatonin-deficient patients attenuate these glycemic oscillations? The objective of this research project is to assess whether melatonin supplementation can reduce glycemic variations throughout the day in patients with T2DM.

## Methods

### Study Design

This prospective, crossover, double-blind, randomized, placebo-controlled study was approved by the ethics committee of the Federal University of Minas Gerais. The study will be conducted at an endocrinology outpatient clinic run by the research physician (WM) in the city of Belo Horizonte, Brazil. Thirty patients will participate in the initial phase of this work. These patients will be randomized to 2 groups: a placebo-washout-melatonin (3 mg) group, which will receive a placebo for 7 days, followed by 7 days without the study medication (washout period) and 7 days of melatonin (3 mg); and group 2, the melatonin-washout-placebo group, which will receive melatonin (3 mg) for 7 days, followed by 7 days without the study medication (washout period) and 7 days of placebo. When 15 patients are allocated to one of the possible intervention orders (placebo followed by melatonin or melatonin followed by placebo), 15 patients will be allocated to the other, not-yet-complete intervention order. Patients will be instructed to use the study medication at 9:00 PM. Patients will receive guidance on diet and physical activity to be followed during these 3 weeks. On the fifth, sixth, and seventh days of the first week and the fifth, sixth, and seventh days of the third week, the patients will use a glucometer to monitor fasting capillary blood glucose before breakfast, 2 hours after breakfast, before lunch, 2 hours after lunch, before dinner, and 2 hours after dinner. The primary outcome analyzed in this study will be the variability of pre- and postprandial glycemia. The result will be compared between 2 periods: with placebo and with melatonin.

### Blinding

**Figure 1:**
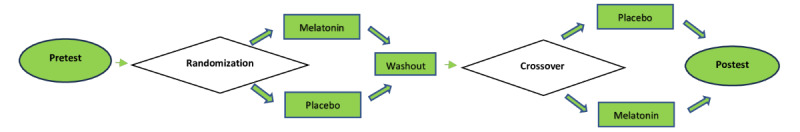
Flowchart.

Medicine bottles containing melatonin or placebo will be numbered 1 and 2, not necessarily in that order. The individual responsible for assigning the numbers will be the sole person with knowledge of which bottles contain melatonin and which contain placebo. All other people involved in this project (patients, medical researchers, statisticians, and assistant personnel) will be blind in relation to the substances. Upon completion of the statistical analysis, the researchers will be informed as to which bottle contained melatonin and which contained placebo. Figure 1 shows a flowchart of the study.

### Glycemic Variability and Crossover Studies

Crossover studies present an advantage over parallel group studies: comparing 2 or more interventions in the same individual reduces possible biases related to differences between groups. Such a design is suitable for chronic diseases and for testing drugs that control a disease but do not cure it. Curing a disease in one period of the study would make it meaningless to use another drug or even a placebo in the following period. When studying an outcome that is influenced by several variables, this design allows for a more reliable comparison with a smaller sample. This is the case for assessing glycemic variability in patients with T2DM. Glycemic variability in patients with T2DM can be influenced by several factors, including medications, duration of diabetes, age, diet, and physical activity. In the evolution of the treatment of diabetes mellitus, there has been an effort to develop drugs that reduce variability in glycemia: dipeptidyl peptidase 4 (DPP-4) inhibitors, GLP1 (glucagon-like peptide 1) analogues, and ultra-slow insulins. Patients with T2DM are treated with a diverse combination of the available drugs. For this reason, pairing groups for a comparison of glycemic variability would be a complex task. Added to this are individual differences in diet and physical activity. Thus, a crossover study is more suitable for the purpose of this work.

### Melatonin and the Carryover Effect in Crossover Studies

Crossover studies should include an adequate washout period, that is, a period in which there is a break from the initial intervention until the beginning of the next intervention. In this work, we propose a period of 1 week for the washout. Questions to examine include whether that time is adequate and whether effects of the initial intervention in the first week remain in the third week. As melatonin has a short half-life (10 to 60 minutes), we believe that its influence will not be present in the third week of the study in the group that uses it initially; a washout period of 1 week thus seems sufficient [20]. Therefore, after a week without using any substance, the initial intervention will not influence the proposed intervention in the third week.

### Glycemic Variability

Glycemic variability refers to fluctuations in blood glucose levels over time. It can be short-term, in self-monitored evaluations of (pre- and postprandial) capillary blood glucose; it can also use the gold standard method, continuous blood glucose monitoring, in which nocturnal blood glucose fluctuations are recorded every 5 minutes [21,22]. There is little correlation between the average variability in glycemic amplitude measured by continuous glucose monitoring and by self-monitoring; however, the latter method can be used for structured assessments of short-term glycemic variability in a day and between different days. The calculation of glycemic variability by this method can also be performed using SDs or even the coefficient of variation. A glycemic variation coefficient of less than 36% is considered level E evidence to separate stable from unstable glycemic control [21]. This measure is considered most effective to assess glycemic variability; however, comparing glycemia between different days is more effective with the mean of daily differences (MODD). This measure is obtained by determining the difference in blood glucose measured at the same time in a 24-hour interval. An additional computer program is used for this measurement, and it cannot be obtained only by continuous capillary blood glucose monitoring devices [21]. In this work, we will use pre- and postprandial capillary glycemia monitoring to determine the glycemic variability of patients with T2DM. In addition to the low cost of the method, we believe that this method gives the work an aspect of a real-life study and therefore greater clinical relevance.

### Inclusion and Exclusion Criteria

Patients will be included in this project according to the following criteria: (1) T2DM duration more than 1 year, (2) age greater than or equal to 40 years, (3) family history of T2DM, and (4) glycohemoglobin between 7% and 10%. Inclusion criterion 1, a T2DM duration of more than 1 year, was chosen because in the first year after diagnosis, patients might not yet have achieved baseline glycemic control. Some patients may have significant improvement in beta-cell glucotoxicity in the first year, with reduced blood glucose levels, and therefore have very wide blood glucose variations from one week to the next because of this improvement. Inclusion criterion 2, the age limit, was established based on the fact that most individuals with T2DM are aged over 40 years. In this way, we avoid including individuals who have autoimmune or genetic diabetes in the study. Item 3 is important because it further homogenizes our patients. We know that T2DM has a strong hereditary component, unlike other types of diabetes. Item 4, limiting the glycohemoglobin value range to 7% to 10%, was established with the objective of homogenizing glycemic variability among our patients, as it is known that glycemic variability increases with an increase in glycohemoglobin.

The following patients will be excluded from this study: (1) pregnant women, (2) patients who have recently (within 3 months) used corticosteroids or other hyperglycemic medications, (3) patients who have conditions that significantly alter glycemic stability (such as renal failure, recent acute coronary syndrome, and other diseases, such as cancer and liver failure). Also excluded will be (4) patients who refuse to participate in this research at any point and (5) patients who have uncontrolled diabetes with symptoms of polydipsia, polyuria, or weight loss that require other measures, such as the use of insulin for glycemic control, in addition to diet, physical activity, and medications already in use. Finally, we will exclude from this study (6) patients at high risk for severe sleep apnea, (7) patients with depression, (8) patients with alternating work shifts, and (9) patients who have epilepsy, a condition that can worsen with the use of melatonin.

Most of these exclusion criteria function to limit conditions that generate very high glycemic variability. It is unlikely that the use of melatonin will be able to reduce glycemic variability induced by corticoids, for example. Patients at high risk for sleep apnea, patients with depression, and patients with alternating work shifts were excluded due to studies that show the negative influence of these conditions on glycemic control. Specific studies with these populations seem more adequate for the purpose proposed here.

### Ethical Aspects

Participants involved in this study will be required to sign an informed consent form that has been drafted in compliance with the Declaration of Helsinki [23]. The wording of the consent form was initially sanctioned, together with the entire project outlined herein, by the ethics committee of the Federal University of Minas Gerais (CAAE: 31990720.7.000.5149). The invitation to participate in this study will be extended during routine consultation at the office of an endocrinology physician (WM). Participants, if they meet the inclusion criteria, will be volunteers. There will be no payment. All research objectives, as well as potential risks pertaining to the use of melatonin, will be explained. We will emphasize, however, that melatonin is a low-risk substance with few side effects. A possible risk is daytime drowsiness, but this is unlikely given the very short half-life of the drug and the low dose used.

### Randomization

Patients will be randomized into two groups. Group 1 will use the substance contained in bottle 1 in the first week, will not use any substance in the second week (the washout period), and will use the substance contained in bottle 2 in the third week. Group 2 will use the substance contained in bottle 2 in the first week, will not use any substance in the second week (the washout period), and will use the substance contained in bottle 1 in the third week.

Patients will be divided into these groups according to the result of rolling a die. Those who roll a number from 1 to 3 will be in group 1, while those who roll a number from 4 to 6 will be in group 2. When the number of participants in a group reaches 15, patients will be automatically allocated to the other group until it includes 15 participants. The project researcher will know which patients will use bottle 1 or 2 in the first and third weeks but will not know which bottle contains placebo and which contains melatonin.

### Interventions

Patients will use melatonin in the first week and placebo in the third week or placebo in the first week and melatonin in the third week, according to the randomization described. On days 5, 6, and 7 of the first and third weeks of the study, patients will measure fasting capillary blood glucose levels 2 hours after breakfast, before lunch, 2 hours after lunch, before dinner, and 2 hours after dinner. Patients will also receive guidance on diet and physical activity to be followed during these 3 weeks. In the second week, patients will undergo a washout period. At the end of the study, the patients will return to the office with their capillary blood glucose results. At this point, they will be asked whether they experienced excessive daytime sleepiness at any point in the study and whether they suspected at any time whether a substance was the placebo or melatonin.

### Procedures

Patients with T2DM will be selected to take part in this study during routine consultations held in an outpatient endocrinology clinic. At the end of the consultation, the endocrinologist, when verifying that the patient meets the inclusion criteria, will ask the patient if they are interested in participating in research on diabetes and melatonin. This question will be followed by reading the informed consent form. All stages of the research will be explained to the patient. This includes the research period, the use of a placebo substance and melatonin, the need to perform capillary blood glucose measurement 6 times a day for 3 days in the first week and 3 days in the third week, the washout period, and the delivery of capillary blood glucose results after the 3 weeks of the project. If the patient agrees to participate in the project, the researcher will ask them to sign the free and informed consent form. A copy of this form will be given to the patient. Next, the researcher will ask the patient to roll a die. Patients who roll a number from 1 to 3 will be in group 1 and will be included in the group that uses the following sequence: bottle 1, washout, bottle 2. Those who roll a number from 4 to 6 will be included in group that uses the following sequence: bottle 2, washout, bottle 1. After allocating the patient to one of the groups, the project researcher will fill in the research record, which will contain the epidemiological, clinical, and laboratory data of the patient, including age, gender, duration of diabetes, history of epilepsy, insulin use (or not), STOP-Bang (snoring, tiredness, observed apnea, high blood pressure, BMI, age, neck circumference, and gender) questionnaire results, weight, height, BMI, abdominal circumference, and glycohemoglobin value. After this step, the patient will be guided on the diet and physical activity regimens to be carried out during the 3 weeks of the project. Patients will be instructed to perform 30 minutes of physical activity 5 times a week during the 3 weeks of the project. After this guidance, patients will receive a glucometer, strips for measuring capillary blood glucose, and lancets to take home. In addition, patients will be given a form to record capillary blood glucose values. At the end of the third week, the patient will return with the blood glucose values.

### Primary and Secondary Outcomes

The primary end point of this study will be the difference in mean variability of the patients’ blood glucose levels between the first and third weeks of the study. The data will be acquired by computing the average of the variance between the total blood glucose levels obtained in these weeks.

As a secondary outcome, we will evaluate the mean disparities in glycemia at each time interval, namely fasting, 2 hours after fasting, before lunch, 2 hours after lunch, before dinner, and 2 hours after dinner. This assessment aims to determine whether the use of melatonin has any discernable impact on blood glucose fluctuation at specific times of the day.

In order to analyze the effect of melatonin after a greater number of days of use, we will perform a specific analysis of the glycemic variability measured on the last day of glycemic measurement in the first and third weeks.

We will also perform a categorical analysis that will take into account the coefficient of variation of blood glucose levels during the use of placebo and during the use of melatonin. We will consider this value to be stable if it is less than 36% and unstable if it is higher than 36%. We will evaluate whether the coefficient of variation was stable or unstable in the week in which melatonin was used.

### Sample Size

The sample size will be 30 patients. As referenced in the literature, when the SD and population frequencies of a given variable are not known, a pretest should be performed with 30 to 40 patients, and data from this group should be considered a population estimate [24]. After collecting blood glucose data for the first and third weeks, the mean glycemic variability and the SD will be calculated so that the sample size can be recalculated. This is necessary because there are no similar studies in the literature. If the recalculated sample size is less than 30, no new participants will be needed and the study will be concluded. If the new sample size is greater than 30, new patients will be recruited until the new, larger sample size is reached.

### Statistical Analysis

If quantitative variables adhere to a normal distribution, as represented by the Gaussian curve, their central tendency will be expressed as mean and their dispersion will be quantified by the SD. If the distribution of these variables does not follow a normal distribution, then their central tendency will be described by the median and their dispersion will be characterized by the 25% and 75% quartiles. The Shapiro-Wilk test will be used to assess normality. Categorical variables will be presented using both absolute frequencies and percentages.

Statistical analyses of normal quantitative variables, both before and after the intervention, will be conducted using a paired 1-tailed *t* test. When the distribution is not normal, the Wilcoxon test will be used. The study will have 2 intervention tails: placebo-washout-melatonin and melatonin-washout-placebo. Comparison of blood glucose levels will be performed between the time when the patients used melatonin and the time they used placebo.

The patients will be compared with one another based on the capillary blood glucose results obtained during the first and third weeks.

The epidemiological, clinical, and laboratory data of the patients will be compared between groups 1 and 2 in order to evaluate whether the randomization process has generated groups that lack statistical differences that may influence the research results.

The significance level will be .05 and the software of choice will be SPSS (version 20.0; IBM Corp).

## Results

**Figure 2:**
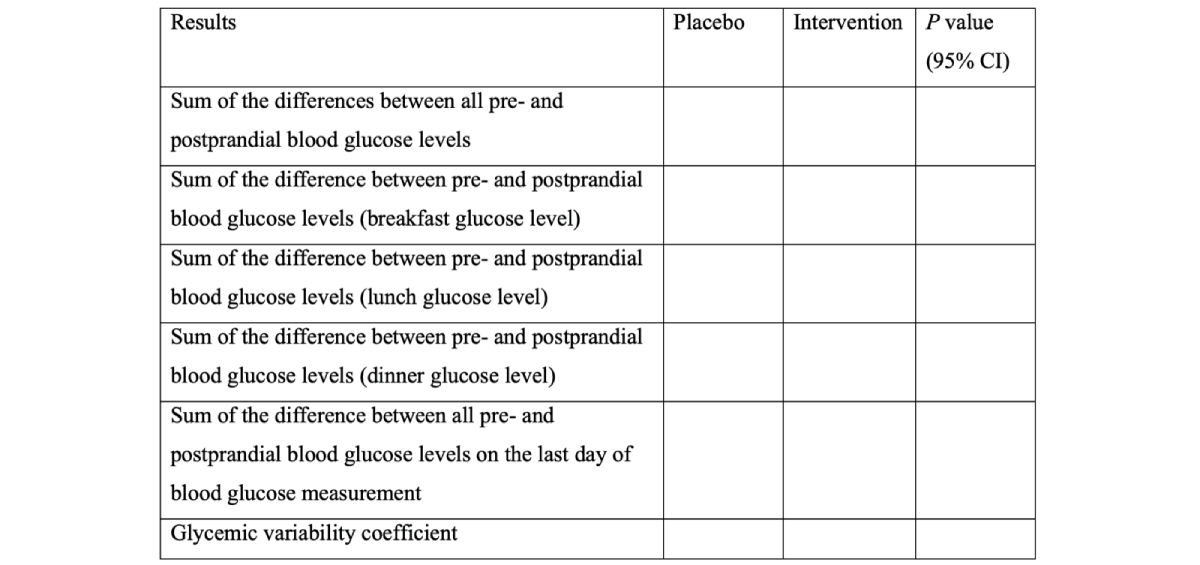
Sample table showing how results will be reported. Unpaired-sample *t* tests or Wilcoxon tests will be used.

Participant recruitment took place between March 2023 to April 2023. In all, 30 participants were eligible and completed the study. Upon selection of these patients and completion of the 3-week research period, a statistical analysis was conducted on the variability of capillary blood glucose levels during the first and third weeks. Subsequently, these data were used to determine the required sample size (n) necessary to draw valid conclusions regarding the proposed research objectives. Furthermore, in addition to the capillary blood glucose values, the data types presented in Table 1 will be scrutinized to ensure the absence of statistical discrepancies between groups 1 and 2. The study was approved by the Brazilian Registry of Clinical Trials (RBR-6wg54rb) on March 7, 2023. Figure 2 shows a sample table of how the results will be presented.

**Table 1:** Epidemiological, clinical, and laboratory data (n=30).

Characteristics	Values
Participants	n
Age (years)	Mean or median
Sex (female)	n, %
Insulin users	n, %
BMI (kg/m^2^)	Mean or median
Abdominal circumference (cm)	Mean or median
Duration of diabetes (years)	Mean or median
STOP-Bang^a^ (score)	Mean or median
Glycohemoglobin (%)	Mean or median

^a^STOP-Bang: snoring, tiredness, observed apnea, high blood pressure, BMI, age, neck circumference, gender.

## Discussion

### Anticipated Findings

The role of melatonin in the glycemic control of T2DM patients has been studied for a number of years [25]. However, studies on the role of melatonin in glycemic variability are not available. Our work does not aim to assess the impact of melatonin on glycohemoglobin levels, but rather its effect on glycemic variability. The hypothesis was tested based on the fact that melatonin, as a hormone, plays a crucial role in regulating circadian rhythms. This notion is logical to us, given that a hormone whose secretion is primarily nocturnal, such as melatonin, governs numerous biological rhythms [26]. Our findings could signify a great advance in the management of T2DM, as it would provide a new therapeutic target: deficient melatonin production in affected individuals. The primary aim of this work is present a new approach toward managing and preventing chronic complications in patients diagnosed with T2DM.

### Limitations

The limitations inherent in this study pertain to an analysis of glycemic variability conducted over a short span: only a few days after intake of melatonin or placebo. The effectiveness of melatonin in reducing glycemic variability might occur with longer use.

A second limitation of this study is derived from the fact that endogenous melatonin peaks in the middle of the night. The exogenous melatonin administered to patients in this study will not reproduce the physiological rhythm of endogenous melatonin.

A third limitation is the fact that patients may have diet and physical activity fluctuations between weeks 1 and 3 of the study, despite the guidance given initially, which might interfere with glycemic variability.

A fourth limitation of this study is the fact that we will not assess the prevalence of the *MTNR1B* mutation, which can determine a poorer glycemic response to melatonin use.

### Conclusions

The study of glycemic variability in patients with T2DM is fundamental for the prevention of chronic complications of the disease. Melatonin is a substance involved in biological rhythms, including those related to glucose control, such as insulin secretion. The existing knowledge that patients with T2DM produce a smaller amount of melatonin motivated us to test the replacement of this hormone as an instrument for greater glycemic stability. As glycemic variability is influenced by several variables, we conclude that a crossover design will be more suitable than a paired-group design for this study to evaluate the role of melatonin in glycemic stability.

## Abbreviations

DPP 4: dipeptidyl peptidase 4
GLP1: glucagon-like peptide-1
MODD: mean of daily differences
STOP-Bang: snoring, tiredness, observed apnea, high blood pressure, BMI, age, neck circumference, gender
T2DM: type 2 diabetes mellitus

## Data Availability

The data sets generated during and/or analyzed during this study will be made available from the corresponding author on reasonable request.
